# Accuracy of Orthodontic Anchor Screw Placement Using a 3D-Printed Surgical Guide

**DOI:** 10.7759/cureus.67431

**Published:** 2024-08-21

**Authors:** Ryosuke Ikenaka, So Koizumi, Heetae Park, Masatoshi Shimura, Kazuhide Seimiya, Shinya Fuchida, Tetsutaro Yamaguchi

**Affiliations:** 1 Department of Orthodontics, School of Dentistry, Kanagawa Dental University, Yokosuka, JPN; 2 Division of Dental Practice Support, Department of Dental Technology, Kanagawa Dental University, Yokosuka, JPN; 3 Department of Education Planning, School of Dentistry, Kanagawa Dental University, Yokosuka, JPN

**Keywords:** computed tomography, 3d printer, root contact, surgical guide, mini screws, orthodontics

## Abstract

Background

Although radiographs and computed tomography (CT) images are reviewed before temporary anchorage device (TAD) implantation, implantation of TADs exactly as planned is difficult. This study aimed to evaluate the accuracy of TAD implantation using an original surgical guide fabricated using cone-beam CT data and computer-aided design software.

Methodology

The study participants included six experienced orthodontists who had implanted ≥20 TADs, and six inexperienced orthodontists who had never implanted a TAD. Maxillary dental typodont models with radiopaque tooth crowns and roots were used. A total of four TADs were implanted on the buccal sides: between the second bicuspid and first molars and between the first and second molars bilaterally. The accuracy of TAD implantation was examined in two groups: in 12 dental typodont models, TAD implantation was performed using a surgical guide (guide group), and in 12 dental typodont models, TAD implantation was performed without a surgical guide (freehand group). All dental typodont models implanted a total of 96 TADs. The TAD position was evaluated using the CT coordinate system and 3D image measurement software. Using the long axis of the TAD as a reference, the distance between the coronal and apical ends of the implanted TAD and those of the planned TAD, i.e., the ideal implantation position, was measured in both groups along the x, y, and z axes. The medians of the values were compared between the groups. Additionally, the presence of root contact was compared between the experienced and inexperienced orthodontists.

Results

On the x-axis, the linear deviations (median) of the coronal and apical ends of the TAD in the freehand group were 1.06 mm and 1.36 mm, respectively. In contrast, in the guide group, the deviations were 0.65 mm and 0.90 mm, respectively, and the difference was statistically significant (p = 0.002 and p = 0.005, respectively). On the y-axis, the deviations in the freehand group were 1.13 mm and 1.08 mm, respectively. In contrast, the deviations in the guide group were 0.71 mm and 0.79 mm, respectively, and only the coronal deviations were significantly different between the groups (p = 0.006). On the z-axis, the deviations in the freehand group were 1.44 mm and 1.86 mm, respectively. In contrast, the deviations in the guide group were 0.75 mm and 1.16 mm, respectively, and the difference was statistically significant (p = 0.006 and p = 0.002, respectively).

Conclusions

The use of a surgical guide allowed for more accurate TAD implantation. Additionally, TAD implantation using a guide prevented root damage.

## Introduction

Temporary anchorage devices (TADs) have several indications and are routinely used worldwide because of their ease of implantation and independence from patient compliance [1]. Recently, several studies have reported the effectiveness and adverse effects of TADs along with measures to prevent such adverse effects [2]. The most frequent complication associated with TADs is failure, with a failure rate of 10-20% [3,4]. Root contact is the most frequent cause of TAD failure [5-7].

Implantation methods with minimal root contact are being investigated [8,9]. Although predicting the exact position and distance between the tooth roots using two-dimensional radiographs is difficult, three-dimensional (3D) dental imaging modalities such as cone-beam computed tomography (CBCT) allow accurate evaluation of the positional relationship of the tooth roots [10]. TADs are most frequently implanted in the inter-root alveolar region between the molars and premolars [11]. However, because the inter-root distance is small and implantation is difficult, surgical guides to avoid root contact have been fabricated using computer-aided design/computer-aided manufacturing (CAD/CAM) techniques using CBCT data, 3D analysis software, and stereolithography apparatus [12,13].

In this study, a simpler and smaller surgical guide than those reported in previous studies was fabricated using CBCT data and CAD software. The guide was designed to be clinically easy to use with any type of TAD implantation driver or shape and allow for proper implantation of the TAD. This study aimed to evaluate the accuracy of TAD implantation using this surgical guide and the effect of the doctor’s skill level on the accuracy.

## Materials and methods

Preparation of dental typodont models

**Figure 1:**
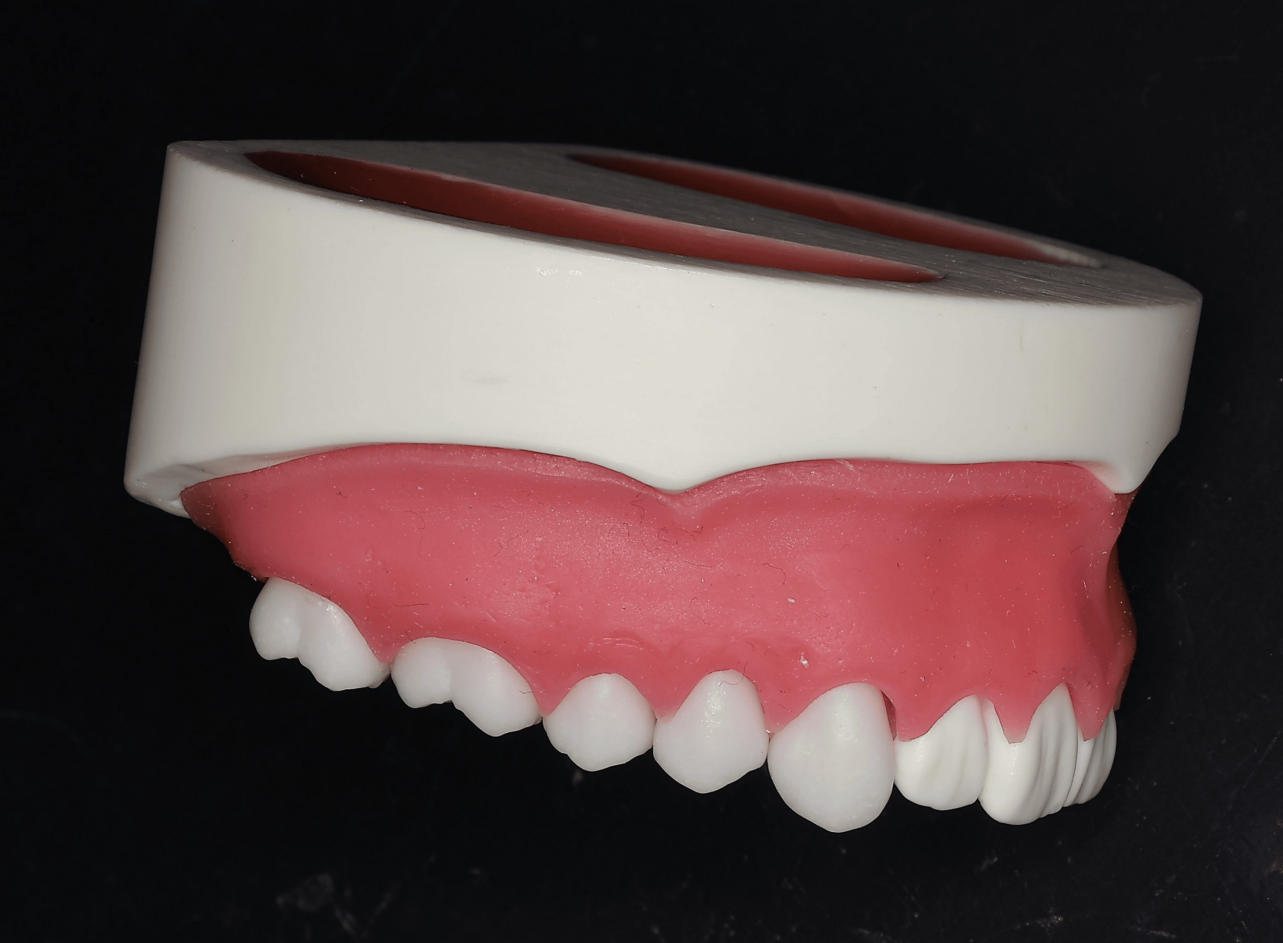
Maxillary dental typodont model for temporary anchorage device implantation. This model consists of plastic teeth, bone, and silicone gingiva.

**Figure 2:**
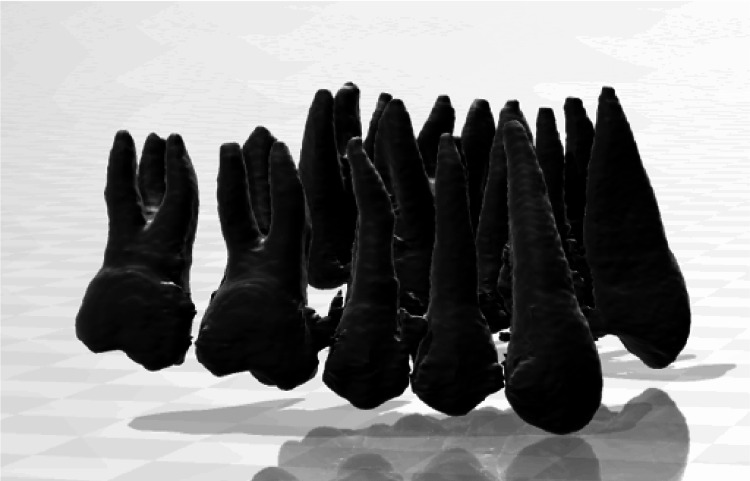
Cone-beam computed tomography image of the maxillary dental typodont model. This image visualizes the crown and roots.

Maxillary dental typodont models with radiopaque tooth crowns and roots were selected (Figure 1) to allow visualization of the positions of the crowns, roots, and TAD on CBCT (Figure 2). This custom-made model was ordered from a dental typodont model-making company (Smile Innovations Alliance, Kyoto, Japan).

Surgical guide design

**Figure 3:**
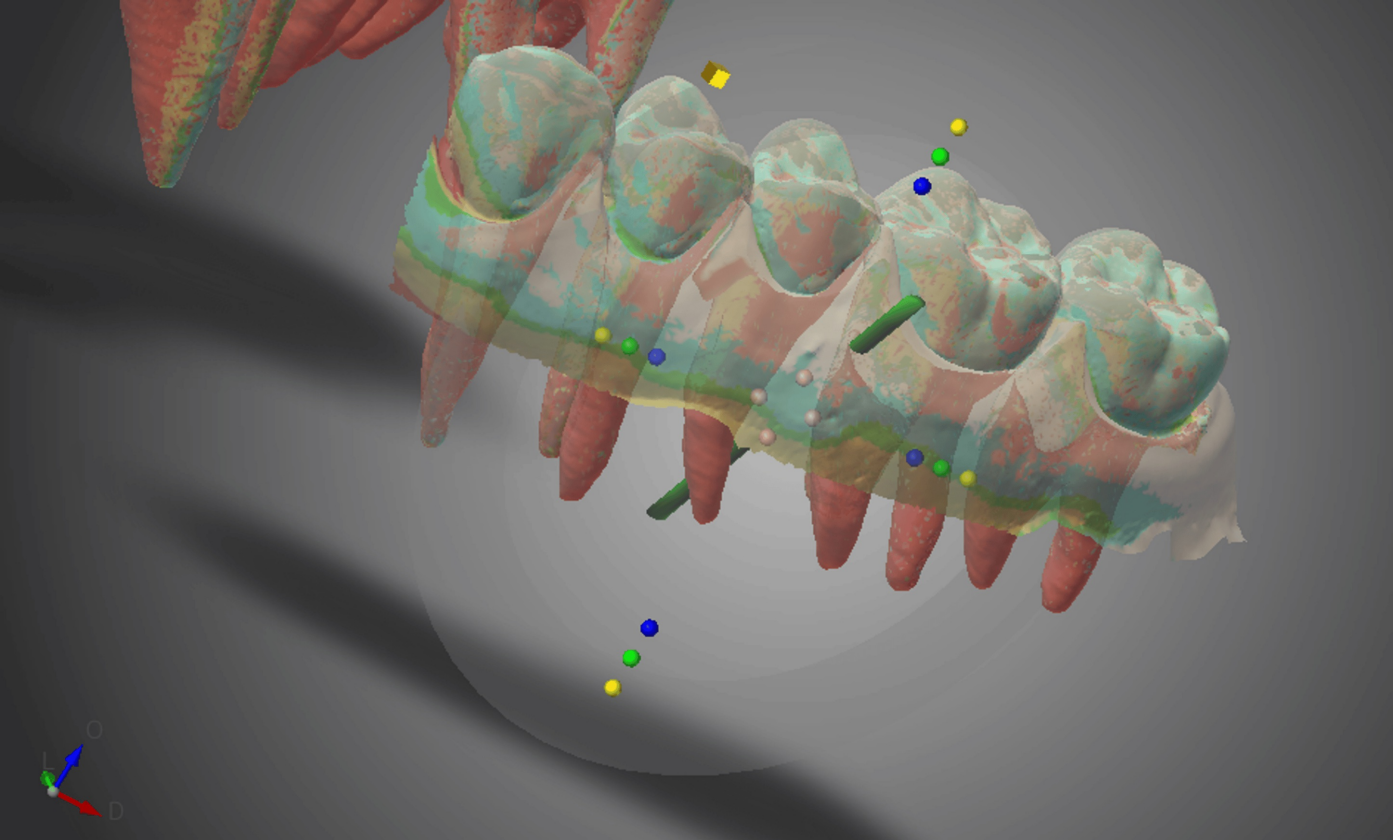
Surgical guide design on 3D device design software. The temporary anchorage device implantation position is then established.

The maxillary dental models were scanned using a CBCT imaging system (Trophypan Smart Sc, Yoshida, Tokyo, Japan), and data for the crowns and roots were extracted from the Digital Imaging and Communications in Medicine (DICOM) data and saved in standard tessellation language (STL) format. Next, an intraoral scanner (iTero® Element™2, Align Technology, CA, USA) was used to scan the tooth crowns and attached gingiva, and the data were saved in STL format. Surgical guides were designed based on this STL data using 3D device design software (Dental Wings, Straumann Group, Montreal, Canada) (Figure 3).

**Figure 4:**
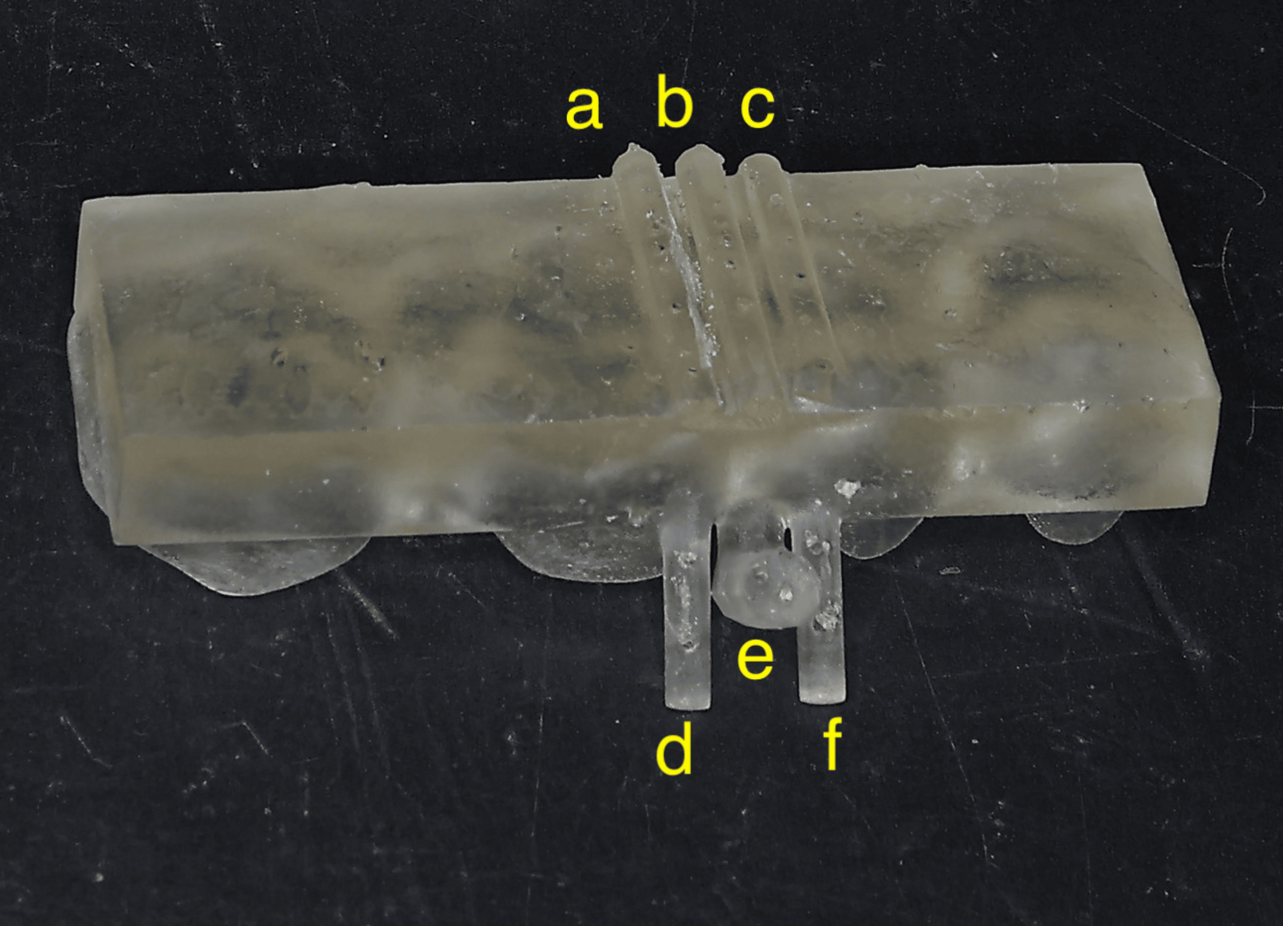
3D-printed surgical guide. a, c: indicator lines; b: guide line; d, f: indicator posts; e: guide post.

**Figure 5:**
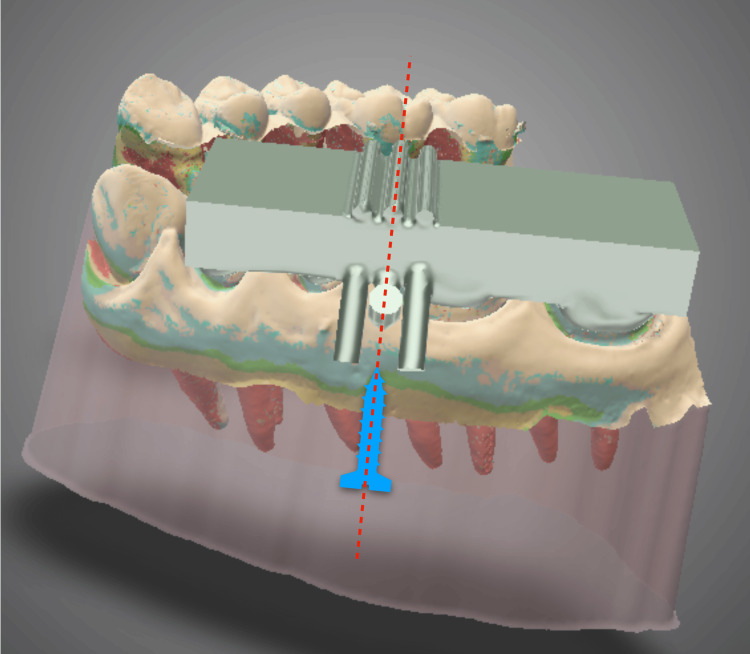
Temporary anchorage device implantation direction using the surgical guide (front view) on 3D device design software. Blue image: Image of temporary anchorage device implantation position. Red dotted line: Direction of temporary anchorage device implantation.

**Figure 6:**
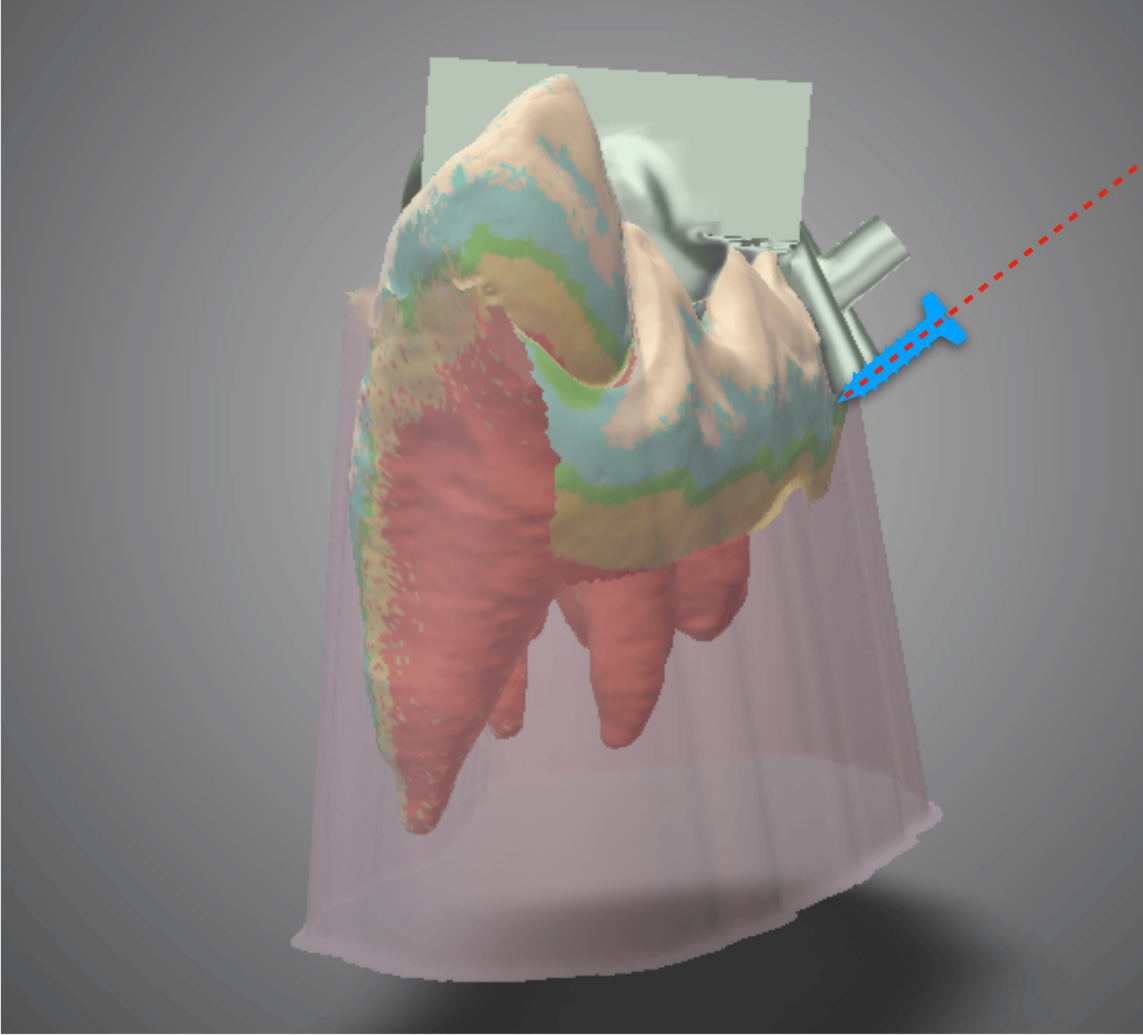
Temporary anchorage device implantation direction using the surgical guide (lateral view) on 3D device design software. Blue image: Image of temporary anchorage device implantation position. Red dotted line: Direction of temporary anchorage device implantation.

Generally, surgical guides are shaped to encompass the tip of the TAD implantation driver; therefore, they tend to be large and must be adjusted according to the tip shape of each TAD driver [14]. The surgical guide reported here was designed with the primary goal of avoiding contact between the TAD and the tooth root. Additionally, it was designed to have the minimum necessary size for controlling the implantation direction. The surgical guide consisted of a grasping plate that covered the occlusal surfaces of approximately four teeth adjacent to the implantation site, a guide line, a guide post that defined the implantation axis in two planes, indicator lines, and indicator posts that clearly indicated the adjacent root margins at the time of implantation (Figure 4). The guide line and guide post were located midway between the indicator line and the indicator post. The midpoint of the line connecting the tips of the two indicator posts at the root apex level on the buccal side was selected as the puncture point (Figure 5). By inserting the TAD parallel to the guide line and guide post from this puncture point, it can be precisely implanted at the planned placement location (Figures 5, 6).

Surgical guide fabrication

Surgical guide design data were input into a CAD/CAM unit (DH Sonic Mighty, Denken High Dental), and surgical guides were fabricated using 3D optical fabrication using the DH Print Splint & Guide (Denken High Dental).

TAD implantation procedure

**Figure 7:**
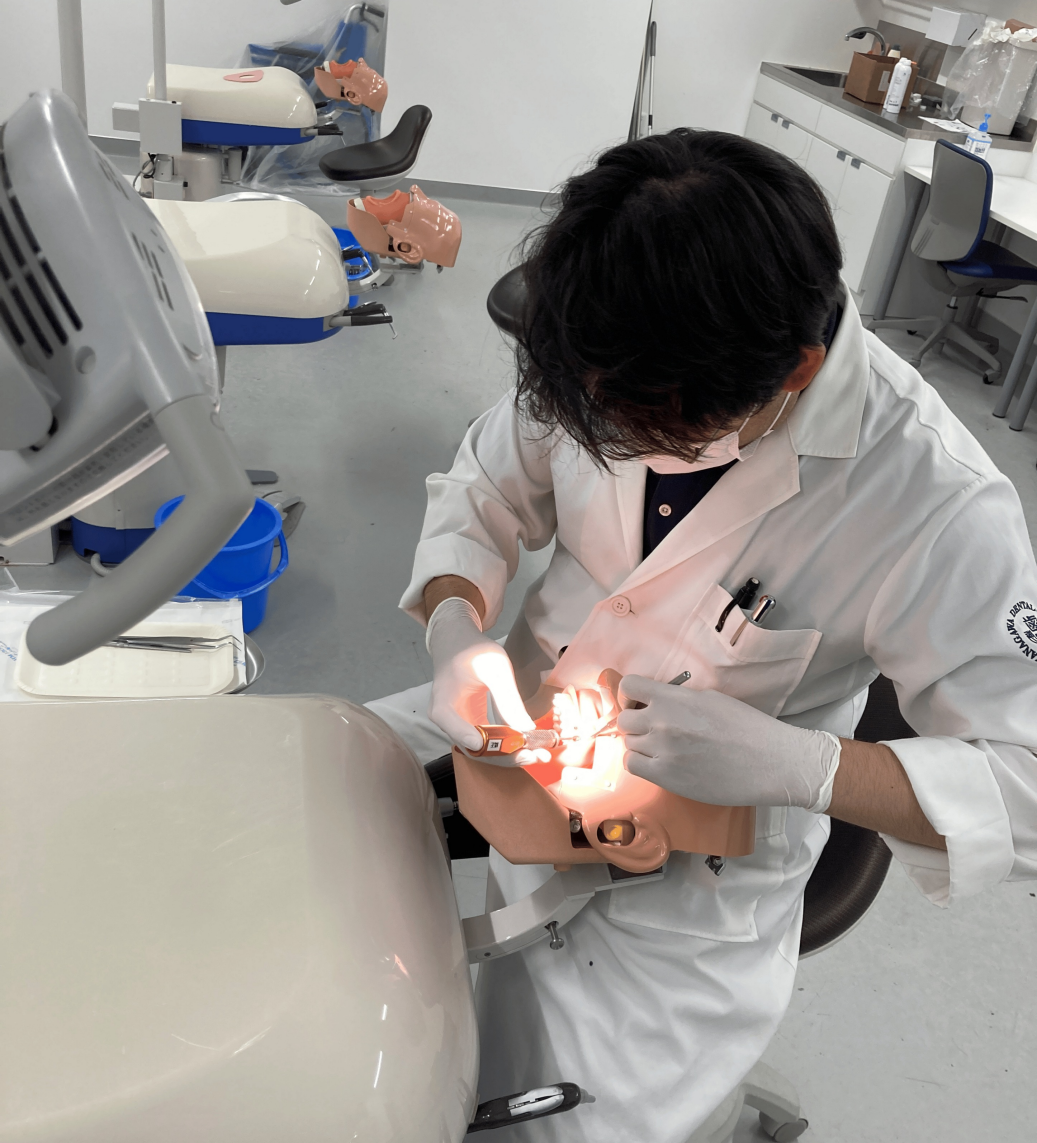
Temporary anchorage device implantation on a phantom model by study participants.

The study participants included six experienced orthodontists who had implanted ≥20 TADs, and six inexperienced orthodontists who had never implanted a TAD. Maxillary dental typodont models were mounted on a phantom model, and the 12 orthodontists implanted 1.4-mm diameter and 8.0-mm long TADs (Bector, Ormco, Tokyo, Japan) using two methods, with and without a surgical guide (Figure 7).

Self-drilling TADs were positioned at four sites on the buccal sides: between the bilateral second bicuspids and first molars and between the bilateral first and second molars.

**Figure 8:**
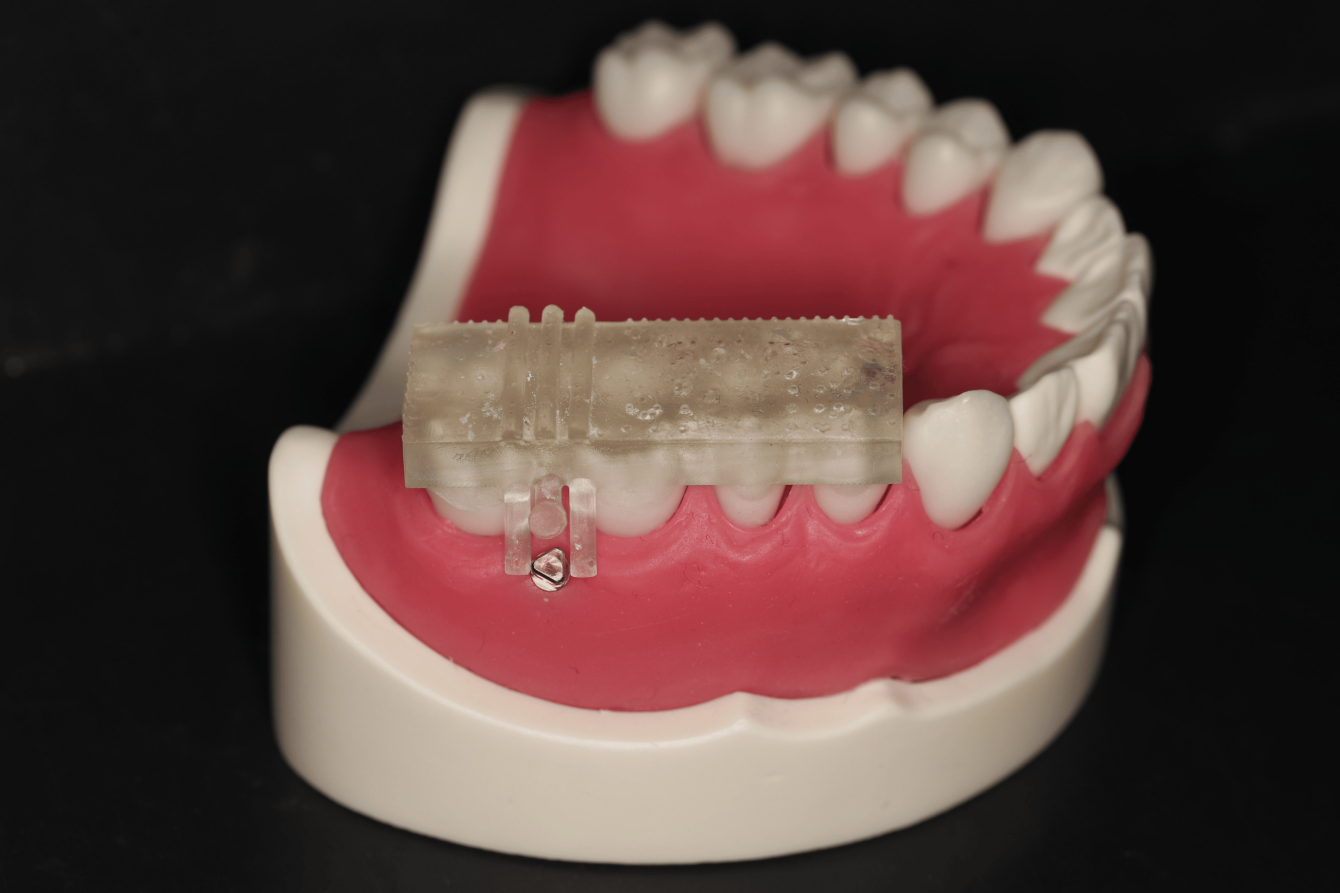
Dental typodont model with temporary anchorage device implantation using a surgical guide.

First, all 12 doctors implanted TADs into the dental typodont models without a surgical guide (freehand group). The position of the root was confirmed on CBCT images before implantation. Next, the TAD was implanted at a 45° angle to the long axis of the first molar by marking 10.0 mm toward the root apex on the line connecting the insertion point and the adjacent molar cusps with a probe. One month later, all 12 orthodontists implanted TADs into the dental typodont models using a surgical guide (guide group) (Figure 8).

Evaluation of TAD placement accuracy

First, a dental typodont model with a TAD implanted in the ideal position using the surgical guide was prepared and CBCT was performed. The obtained DICOM data were used as the reference image.

**Figure 9:**
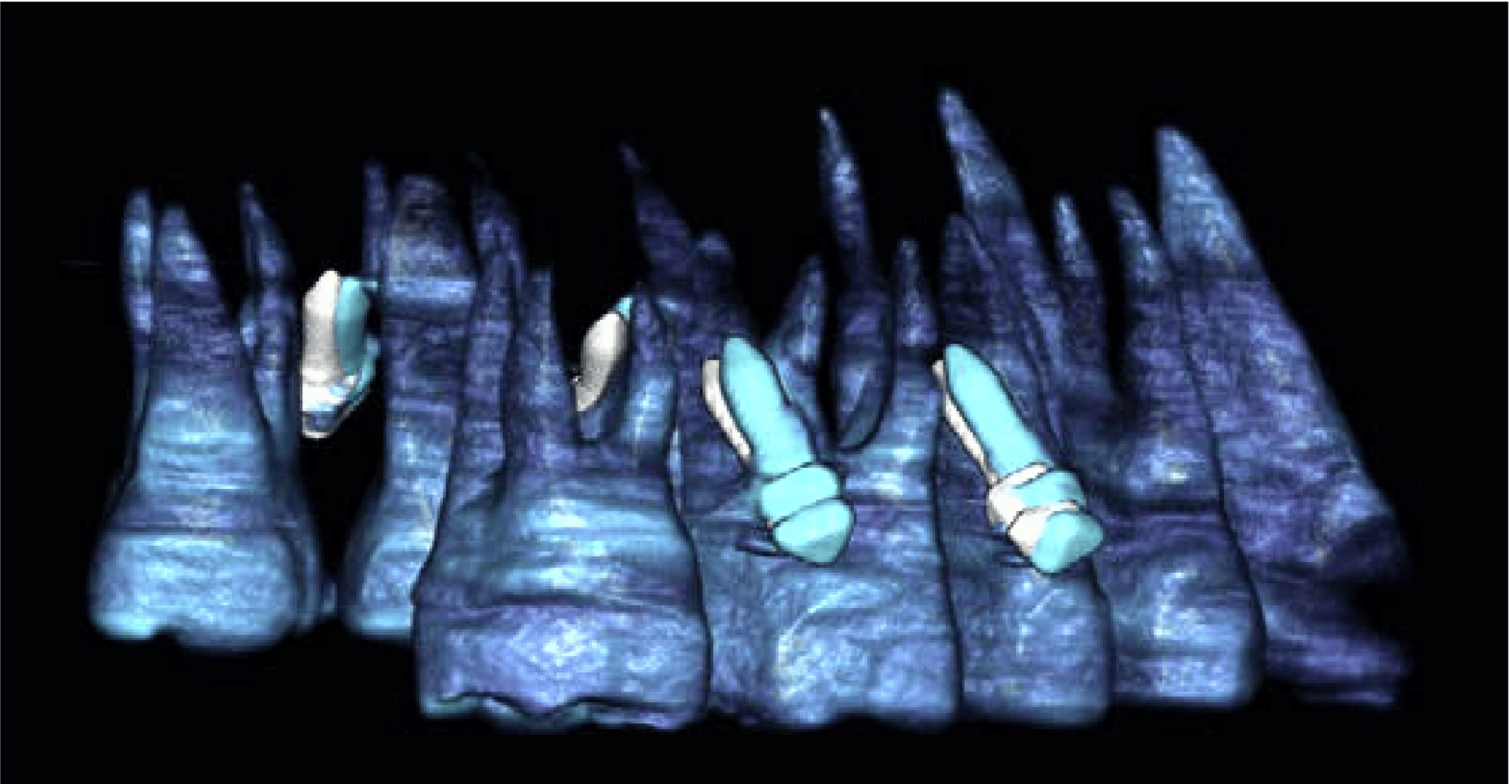
The temporary anchorage device implantation data superimposed on the reference data to determine the difference from the ideal position on 3D image analysis software. White image: The ideal temporary anchorage device implantation position (reference image). Blue image: The actual temporary anchorage device implantation position.

Next, CBCT was performed for the dental typodont models with TADs implanted by the study participants. The DICOM data were superimposed on the reference image using 3D image analysis software (Invivo6, Anatomage, San Jose, CA, USA) (Figure 9).

**Figure 10:**
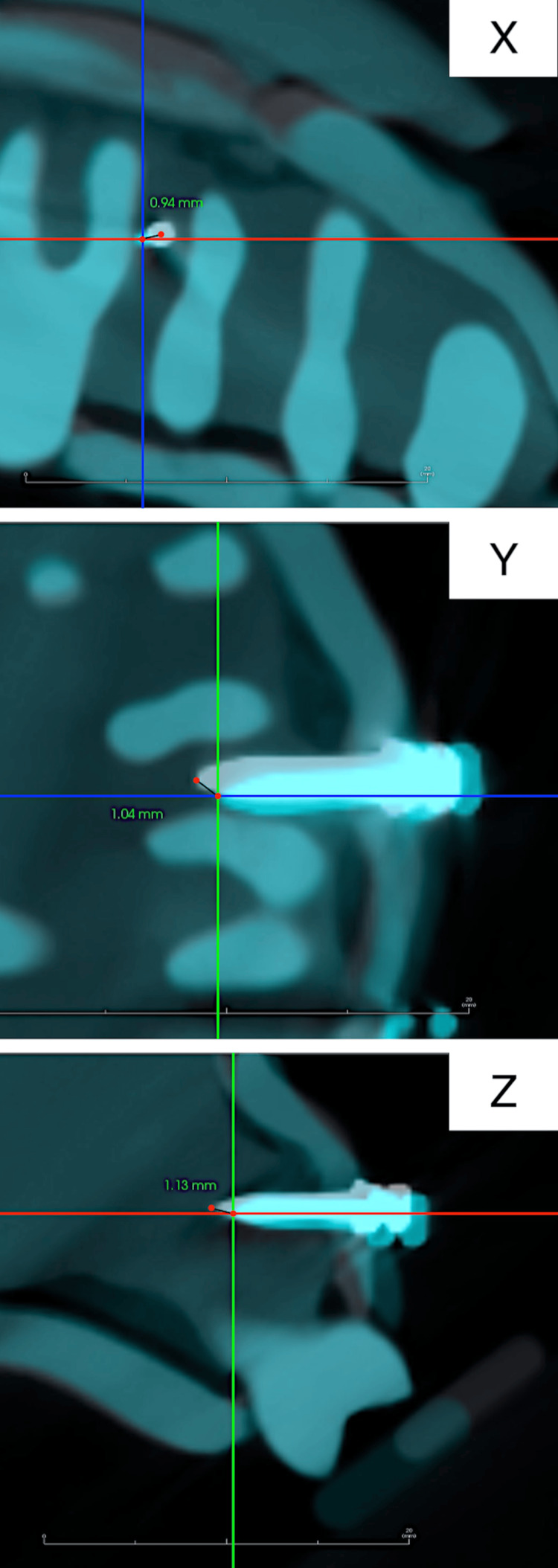
Measurement of the distance between the ideal and actual positions of the temporary anchorage device apex from the x-, y-, and z-axes on 3D image analysis software. The intersection of the two lines represents the actual temporary anchorage device implantation position, which corresponds to the temporary anchorage device apex. White image: The ideal temporary anchorage device implantation position. Blue image: The actual temporary anchorage device implantation position.

In the CBCT coordinate system, the x-axis represents the long-axis direction of the TAD, the y-axis represents the mesiodistal direction, and the z-axis represents the tooth axis direction. The distances of the coronal and apical ends of the ideal and actual implantation positions of the TADs from the three axes were measured (Figure 10), and the differences were compared between the groups.

Statistical analysis

The mean of the respective distances on the x-, y-, and z-axes at the coronal and apical ends of the TAD was used for the analysis of the accuracy of implantation.

The normality of the six deviations between the planned and actual TAD positions in the freehand and guide groups was evaluated using the Shapiro-Wilk test. As the data did not meet the criteria for normality, the median, interquartile range, and minimum and maximum values were calculated and compared between the freehand and guide groups using the Wilcoxon signed-rank test.

As this study was designed to test a specific hypothesis, the required sample size was not determined before conducting the statistical analysis. Statistical analyses were performed using SPSS Statistics (version 23.0; IBM Corp., Armonk, NY, USA), and statistical significance was set at p-values <0.05.

## Results

On the x-axis, the linear deviations (median and interquartile range) of the coronal and apical ends of the TAD in the freehand group were 1.06 (0.95-1.49) mm and 1.36 (1.18-2.14) mm, respectively. In contrast, in the guide group, the deviations were 0.65 (0.53-0.94) mm and 0.90 (0.81-1.19) mm, respectively, and the difference was statistically significant (p = 0.002 and p = 0.005, respectively).

On the y-axis, the deviations in the freehand group were 1.13 (0.97-1.32) mm and 1.08 (0.84-1.31) mm, respectively. In contrast, the deviations in the guide group were 0.71 (0.46-0.96) mm and 0.79 (0.72-1.01) mm, respectively, and only the coronal deviations were significantly different between the groups (p = 0.006).

On the z-axis, the deviations in the freehand group were 1.44 (1.03-1.72) mm and 1.86 (1.51-2.25) mm, respectively. In contrast, the deviations in the guide group were 0.75 (0.62-1.10) mm and 1.16 (0.87-1.38) mm, respectively, and the difference was statistically significant (p = 0.006 and p = 0.002, respectively) (Table 1).

**Table 1:** Deviation of actual TAD position from the planned position. All values are in mm. Wilcoxon signed-rank test. TAD: temporary anchorage device

		Coronal x	Coronal y	Coronal z	Apical x	Apical y	Apical z
Freehand group (total 48 TADs)	Minimum	0.85	0.76	0.86	0.99	0.78	1.33
	25%	0.95	0.97	1.03	1.18	0.84	1.51
	50% (median)	1.06	1.13	1.44	1.36	1.08	1.86
	75%	1.49	1.32	1.72	2.14	1.31	2.25
	Maximum	2.30	1.43	2.21	2.41	1.89	2.72
Guide group (total 48 TADs)	Minimum	0.28	0.36	0.37	0.62	0.45	0.62
	25%	0.53	0.46	0.62	0.81	0.72	0.87
	50% (median)	0.65	0.71	0.75	0.90	0.79	1.16
	75%	0.94	0.96	1.10	1.19	1.01	1.38
	Maximum	1.13	1.03	1.30	1.29	1.18	1.64
P-value		0.002	0.006	0.006	0.005	0.065	0.002

Root contact was observed in 18.8% of the TADs in the freehand group, whereas no TADs in the guide group exhibited root contact. Furthermore, root contact was not observed in the guide group, irrespective of the operator’s experience with TAD implantation (Table 2).

**Table 2:** Comparison of root contact between the freehand and guide groups. TAD: temporary anchorage device

	Freehand group (total 48 TADs)	Guide group (total 48 TADs)
Orthodontists with TAD implantation experience	4	0
Orthodontists without TAD implantation experience	5	0
Frequency of root contact	18.8%	0%

## Discussion

Direct implantation of TADs without a surgical guide relies on the doctor’s senses and is associated with a risk of damage to adjacent tooth roots owing to the narrow field of vision. Therefore, the anatomy of the roots and alveolar bone must be evaluated in three dimensions using CBCT before TAD implantation [8]. However, root contact occurs in some cases despite understanding the anatomy. In this study, the TAD position was determined based on 3D images, and a surgical guide was fabricated to eliminate root contact as much as possible.

The surgical guide fabricated in this study was a simple rectangular resin plate covering four teeth with a guide line (implantation axis) (Figure 6). This design has not yet been reported in the literature. Because of the simple design, the time required to fabricate the surgical guide can be reduced.

Moreover, the guide can be used with any type of TAD driver or TAD because its TAD embedding entrance is not affected by the form of the TAD driver. This surgical guide can be adapted to different scenarios to suit the doctor and patient.

In this study, no root contact was observed in the guide group despite the different levels of experience of the 12 study participants. This suggests that the use of a surgical guide can reduce operator-related errors. The apical position of the TAD on the y-axis was not significantly different between the freehand and guide groups. This was thought to be due to variations in the planned apical position of the TAD. Perhaps, even with the use of a surgical guide, visual discrepancies occurred because of the skill level of the operator, resulting in slight differences in the implantation position. However, because no root contact was observed in the guide group, this result did not appear to be particularly problematic.

In the freehand group, root contact was observed in 18.8% of the TADs. This percentage is high considering that the implantation site was confirmed in advance using CBCT. However, root contact was not observed in TADs implanted using the surgical guide fabricated in this study. Furthermore, both experienced doctors who had implanted >20 TADs and inexperienced doctors who had never implanted a TAD achieved successful TAD implantation without root contact. This indicates that the surgical guide is effective, even for inexperienced doctors.

Various factors have been examined for TAD failure, but root contact is the most important [5,6]. Particularly, a longer root contact of the TAD increases the risk of failure [7]. In this study, root contact was not observed when the surgical guide was used; therefore, in clinical practice, the success rate of TADs implanted using this guide is expected to be higher than that with freehand implantation.

Angled placement is strongly recommended to improve the accuracy of TAD implantation in the intermolar region, particularly in the maxilla [15]. In addition, implantation at an angle of 45° to the tooth axis is considered ideal [16]. This ideal positioning is crucial for controlling the angle of implantation in clinical practice. In cases where proximity to the maxillary sinus makes large-angle implantation impractical, the implantation angle in the guide can be adjusted to a smaller value. This adjustment allows for the implantation of the TAD at an optimal angle in a variety of clinical scenarios.

In this study, self-drilling TADs were used; however self-tapping TADs can also be used because predrilling is commonly performed for self-tapping TADs to prevent an excessive increase in the embedding torque [17]. However, the success rates of self-drilling and self-tapping TADs are similar, and evidence to suggest that self-tapping TADs prevent an excessive increase in the insertion torque and are effective in preventing failure is insufficient [18]. Root contact is associated with insertion torque, which, in turn, is related to the failure rate, as the insertion torque increases owing to contact with the tooth root during TAD implantation [19].

This study had some limitations. Because the surgical guide fabricated in this study is simple, small, and easy to fabricate, it cannot be used to fix the TAD driver and relies on visual assessment. This limits the accuracy of TAD implantation. However, this study demonstrated that the accuracy of the guide for avoiding root contact can be guaranteed. Visually confirming the guide line and guide post and manually adjusting the implantation direction can provide a training effect that improves the practitioner’s skills. In this study, TAD implantation was performed using a dental typodont model; therefore, the long-term prognosis could not be evaluated. Application of the surgical guide used in this study in clinical practice may provide useful information on the accuracy of TAD implantation and the long-term prognosis.

## Conclusions

In this study, TAD implantation accuracy was significantly different between the freehand and guide groups. This indicates that the guide allowed more accurate TAD implantation. Furthermore, no damage to the tooth roots was observed in the guide group. This suggests that the surgical guide used in this study allows TAD implantation in the ideal position, regardless of the doctor’s experience.
